# High-throughput isolation of giant viruses using high-content screening

**DOI:** 10.1038/s42003-019-0475-6

**Published:** 2019-06-19

**Authors:** Rania Francis, Yusuke Ominami, Jacques Yaacoub Bou Khalil, Bernard La Scola

**Affiliations:** 1 Institut Hospitalo-Universitaire Méditerranée-Infection, Marseille, 13385 France; 2 Aix-Marseille Université, Institut de Recherche pour le Développement (IRD), UMR Microbes Evolution Phylogeny and Infections (MEPHI), Marseille, 13385 France; 3 Hitachi High-Technologies Corporation, Nanotechnology Solutions Business Group, 24-14 Nishi-shimbashi 1-chome, Minato-ku, Tokyo 105-8717 Japan

**Keywords:** Water microbiology, Virus-host interactions

## Abstract

The race to discover and isolate giant viruses began 15 years ago. Metagenomics is counterbalancing coculture, with the detection of giant virus genomes becoming faster as sequencing technologies develop. Since the discovery of giant viruses, many efforts have been made to improve methods for coculturing amebas and giant viruses, which remains the key engine of isolation of these microorganisms. However, these techniques still lack the proper tools for high-speed detection. In this paper, we present advances in the isolation of giant viruses. A new strategy was developed using a high-throughput microscope for real-time monitoring of cocultures using optimized algorithms targeting infected amebas. After validating the strategy, we adapted a new tabletop scanning electron microscope for high-speed identification of giant viruses directly from culture. The speed and isolation rate of this strategy has raised the coculture to almost the same level as sequencing techniques in terms of detection speed and sensitivity.

## Abstract (web summary)

Rania Francis et al. develop a new strategy for the identification and isolation of giant viruses in real time by using optimized algorithms targeting infected amebas in co-cultures. This new approach, which does not require sequencing techniques, is highly sensitive and 100-fold faster than other high throughput procedures.

## Introduction

Tracking giant viruses has become a large-scale scientific challenge since their discovery, as these viruses challenge the traditional definition of a virus^1,2^. It has been suggested that these viruses could represent a new domain of life, and a new Megavirales order has been recently proposed for their classification^3,4^. Giant viruses have reportedly been isolated from a large diversity of environmental samples, such as Pithovirus and Mollivirus, which were isolated from 30,000-year-old permafrost^5,6^, Tupanviruses, isolated from Soda Lake and the Deep Ocean in Brazil^7^ and many others that have been recovered from various environments^8–12^. Later, several metagenomic studies suggested the ubiquity of giant viruses in the environment and even in humans^13–17^. Many bioinformatics and in silico studies yielded referenced databases related to giant viruses, and a new viral subfamily, Klosneuvirinae, has been proposed solely from metagenomic data^18^. In only 15 years, the status of giant viruses has changed from odd to the richest oceanic microbial population, including deep sea^19,20^ and a hidden diversity of these viruses has been found in soil^21^. Although metagenomics can be used to explore new giant viruses, having access to the viral particles remains crucial since their isolation enables detailed studies of the detected virus and the interactions with its host.

The natural hosts of giant viruses are usually unknown, as few viruses have been isolated with their original hosts^22–24^. Most isolates have been obtained by coculture on adherent amebas, mostly *Acanthamoeba polyphaga* and *Acanthamoeba castellanii*^25–28^. In parallel, other studies have shown that the expansion of the amebic host panel has led to new and more diversified isolates, where *Vermamoeba vermiformis* allows the multiplication of Faustovirus and other closely related viruses^29,30^ that are considered specific to this amebic host.

Regardless of all isolated giant viruses, traditional isolation techniques remain fastidious, time consuming and operator dependent, thus limiting the possibility of broadening the spectrum of cellular hosts and sample diversity. Therefore, the need arises for automated methods capable of overcoming these limitations. Many attempts and strategies have been developed to improve giant virus isolation techniques^28^. Isolation on agar plates was first developed by Boughalmi et al., allowing the detection of amoebal lysis with the naked eye^31^. This technique, although efficient, remained fastidious and time consuming with a high risk of cross contamination between samples. This method was also limited to protozoa growing on agar surfaces. Recently, Bou Khalil et al.^32^ proposed a new system for the detection of amoebal lysis using flow cytometry. This technique allowed the use of highly motile protozoa as host cells. When coupled with sorting, this technique also allowed the separation of viral mixtures^33^. Despite these improvements, coculture and bench work are still lagging behind the speed of metagenomics and bioinformatics tools. However, we are still far from optimizing an isolation strategy that equals the standards and speed of the new sequencing technologies. In this work, we introduce new tools for high-throughput automated detection and isolation of giant viruses using a new generation of microscope for live screening and big data analysis followed by high-speed scanning electron microscopy for preliminary morphological characterization of isolates. This strategy speeds up the isolation procedure 100-fold compared to previous high-throughput procedures.

## Results

### Detection of infected ameba

During the developmental stage, the viability of the ameba, *Acanthamoeba castellanii* strain Neff 30010 (*A. castellanii* Neff), *Vermamoeba vermiformis* strain CDC19 (*V. vermiformis*), *Acanthamoeba polyphaga* strain Linc AP-1 (*A. polyphaga*), and *Acanthamoeba castellanii* strain Douglas 50370 (*A. castellanii* Douglas), was monitored for 5 days for possible toxicity due to SYBR Green. No toxic effect (mortality or encystment) was observed during this period. All stained amebas had the same morphology and concentration as the negative controls without SYBR Green. *A. castellanii* Neff, *A. castellanii* Douglas and *A. polyphaga* showed excellent stable signals over time. However, *V. vermiformis* did not show sufficient SYBR Green uptake, and only a small portion of the cells were stained, even at higher dye concentrations. We tested different DNA stains to find a suitable stain for this ameba. NucBlue staining appeared to be the most convenient for *V. vermiformis*, with the least toxic effect. We then tested the NucBlue on the other amebas, and the results were similar to the SYBR Green staining, but with slightly more background noise. Finally, we used SYBR Green staining for *A. castellanii* Neff, *A. castellanii* Douglas, and *A. polyphaga* and NucBlue for *V. vermiformis*.

The screening strategy adapted to the different time points seemed to be the most convenient way to compromise between screening time, photobleaching of the dye and coverage of the entire well without losing sensitivity. The configuration of the detection was based on the signal intensity of the negative control. Signal variation was considered a feature indicating the presence of infection. We observed a significant increase in the average and total intensities of SYBR Green and NucBlue in infected cells compared to the negative control (*p* value of 0.008). The images showed a fluorescent spot of increased brightness within the cytoplasm at different times of infection corresponding to cytoplasmic viral replication. This signal vanished after the cell burst. In the negative control, the fluorescence signal intensity was diffuse in the cytoplasm and not only localized at the level of the nucleus due to unspecific SYBR Green uptake. Furthermore, amebas started to lose their trophozoite shape following the infection and became rounded. These morphological changes resulted in a significant decrease in the shape index of infected amebas (*p* value of 0.02). As a result, both the fluorescence signal increase and the shape index decrease were considered a positive signal for infection, which allowed us to detect the presence of giant viruses at an early stage prior to host cell lysis. Encysted amebas were easily differentiated from infected amebas based on a specific profile consisting of rounded cells without any increasing fluorescence signal. Moreover, amoebal cell lysis was detected by the drop in the total cell count and the loss of the fluorescence signal intensity at the late stage of infection. We noticed a slight nonsignificant photobleaching of the dye after several scans.

### System sensitivity

Regarding the system sensitivity, detection of infection was possible at a low viral load (MOI of 0.001 for *Acanthamoeba polyphaga* Mimivirus (APMV) on *A. castellanii* Neff. A very high sensitivity was observed, where the software was able to detect an infection when only 3% of the cells were infected.

### Study of viral infectivity and fitness on a selected panel of amebas

The success of the developmental stage and the reproducibility of the results allowed us, using this fully automated imaging system, to study giant viral infectivity and fitness on the selected panel of amebas. The infectivity of each giant virus targeting a specific host was detected successively according to the signal strength and shape index, where there were different viral profiles or signatures regarding signal strength and timing of cell lysis.

**Fig. 1:**
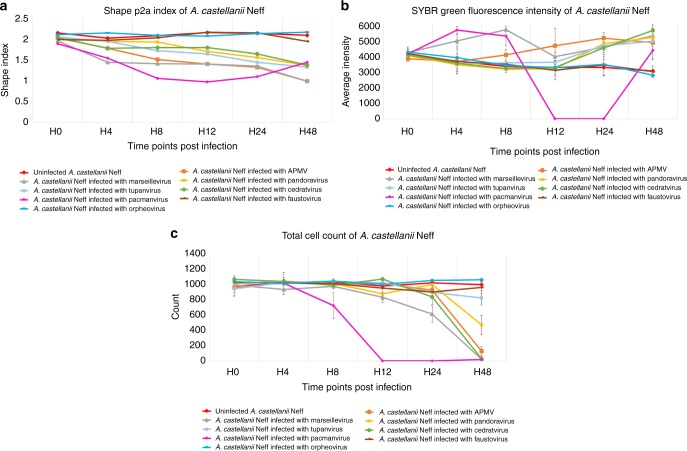
Giant virus infectivity profiles and signatures in *A. castellanii* Neff analyzed by high-content screening. Targeted algorithms for image analysis were configured on the negative control *A. castellanii* Neff stained with SYBR Green. The mean values are represented for each parameter (*n* = 3 independent experiments). Error bars represent standard deviations. In addition, *p*-values were generated for each parameter. **a** Cell Shape P2A Index (*p*-value of 0.02). **b** SYBR Green Average Intensity *p*-value of 0.008 and **c** total cell count (*p*-value of 0.005)

**Fig. 2:**
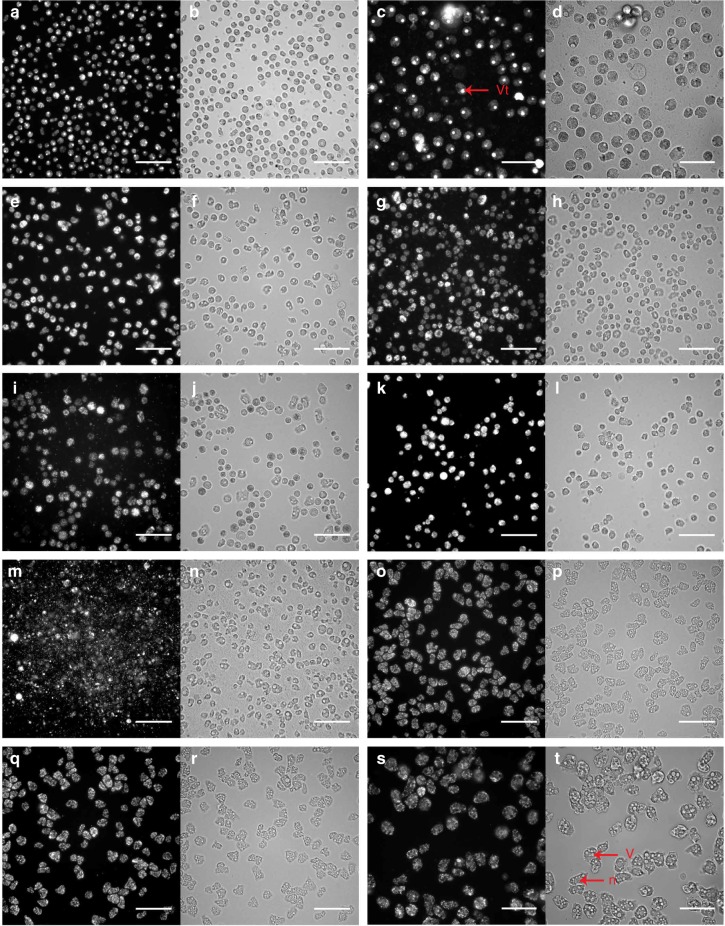
Specific feature detection of infected *A. castellanii* Neff by high-content screening. All cells are stained with SYBR Green. The scale bars indicate 100 µm. **a** SYBR Green channel for APMV at 24 h pi. **b** Brightfield channel for APMV at 24 h pi. **c**, **d** higher magnification of APMV at 24 h pi (scale bar indicates 50 µm). Bright spots representing the viral factory (vf) are well differentiated from the nucleus (n) and the vacuoles (v). **e**, **f** Marseillevirus T19 at 10 h pi. **g**, **h** Pandoravirus massiliensis at 18 h pi. **i**, **j** Tupanvirus Deep Ocean at 24 h pi. **k**, **l** Pacmanvirus at 6 h pi. **m**, **n** Cedratvirus at 20 h pi. **o**, **p** Faustovirus E12 at 48 h pi. **q**, **r** Orpheovirus IHUMI - LCC2 at 48 h pi. **s**, **t** negative control *A. castellanii* Neff at 48 h pi at high-magnification (scale bar indicates 50 µm)

First, for *A. castellanii* Neff infected with APMV, cells began rounding at 8h post infection (pi), which coincided with the formation of a bright fluorescent spot inside the cytoplasm representing the Mimivirus viral factory. These changes resulted in a decrease in the shape index (Fig. 1a) and an increase in fluorescence intensity to its maximal value at 24 h pi (Fig. 1b) when viral multiplication was at its highest. Higher magnification (×40) provided more insights regarding the localized signal of the viral factory (Fig. 2a–d). Consequently, the total cell count decreased following cell host lysis 48 h pi (Fig. 1c). Similar results were observed for Marseillevirus T19, Pacmanvirus, Tupanvirus Deep Ocean, Pandoravirus massiliensis and Cedratvirus, with each having a specific profile (Figs. 1 and 2). Pacmanvirus showed a remarkable viral fitness and infectivity toward *A. castellanii* Neff, *A. castellanii* Douglas and *A. polyphaga*, where it showed a dramatic cell burst at 12 h pi. Another signal intensity increase was observed at 48 h pi, which was due to late reinfection (Fig. 1b). However, no effect was observed regarding the infection of *A. castellanii* Neff with Faustovirus E12 and Orpheovirus IHUMI-LCC2 (Figs. 1 and 2). Similar results were found for the infectivity of *A. polyphaga*, where a cytopathic effect was observed when infected with APMV, Marseillevirus, Pandoravirus, Pacmanvirus and Tupanvirus. Cells remained intact when exposed to Faustovirus, Orpheovirus and Cedratvirus. However, for *A. castellanii* Douglas, a cytopathic effect was only detected when infected with APMV, Marseillevirus, Pacmanvirus and Tupanvirus. No signs of infection were detected with the other giant viruses tested. Finally, regarding the infectivity of *V. vermiformis*, only Tupanvirus, Faustovirus and Orpheovirus caused an infection. Cells remained intact when exposed to the other giant viruses. In addition, the nonlytic Clandestinovirus was only able to infect *V. vermiformis*, for which an increasing fluorescence signal was observed starting 24 h pi, and cells remained rounded for 5 days in the starvation medium (Supplementary Fig. 1). As a result, we managed to create a specific profile for each giant virus and its associated amebas (Table 1).

**Table 1:** Results of the giant virus infectivity assay on different amebas

	*A. castellanii* Neff	*A. polyphaga*	*A. castellanii* Douglas	*V. vermiformis*
APMV	+	+	+	−
Marseillevirus T19	+	+	+	−
Pandoravirus massiliensis	+	−	−	−
Faustovirus E12	−	−	–	+
Tupan Deep Ocean	+	+	+	+
Cedratvirus	+	−	−	−
Orpheovirus IHUMI-LCC2	−	−	−	+
Pacmanvirus	+	+	+	−
Clandestinovirus	−	−	−	+

(+) cell lysis and (−) intact cells

### Artificial sample detection

To validate our new technique, a blind test was performed. We cultured a total of 12 samples out of which 5 were artificially contaminated with giant viruses. As a result, we were able to detect all 5 artificially contaminated samples. The detection of infection was possible at the level of the subculture and even at the primoculture step for some of the samples. At 0 h pi, all samples showed a SYBR Green/NucBlue intensity similar to that of the negative control. No morphological changes were observed at this time point. An increasing fluorescence intensity was observed a few hours pi along with the multiplication of the giant viruses. In parallel, a decreased shape index was observed due to the rounding of infected cells, while the negative control showed no substantial changes. These parameters allowed for the early detection of infection prior to host cell lysis.

### Xenic culture model

In order to test the possibility of using amebas feeding on bacteria, a xenic culture model was realized, for which we cultured *A. castellanii* Neff with live *Enterobacter aerogenes* and infection was carried out with APMV. The results showed a fluorescence profile similar to that of the axenic culture of *A. castellanii* Neff infected with APMV. No increased fluorescence signal related to the bacteria was observed in the uninfected amebas, and this signal increased only when they were infected with APMV. In addition, bacteria stained with SYBR Green were visible outside of amebas but could be excluded algorithmically because of their small size (Supplementary Fig. 2).

### Validating the correlation between fluorescence intensity signal and viral multiplication

**Fig. 3:**
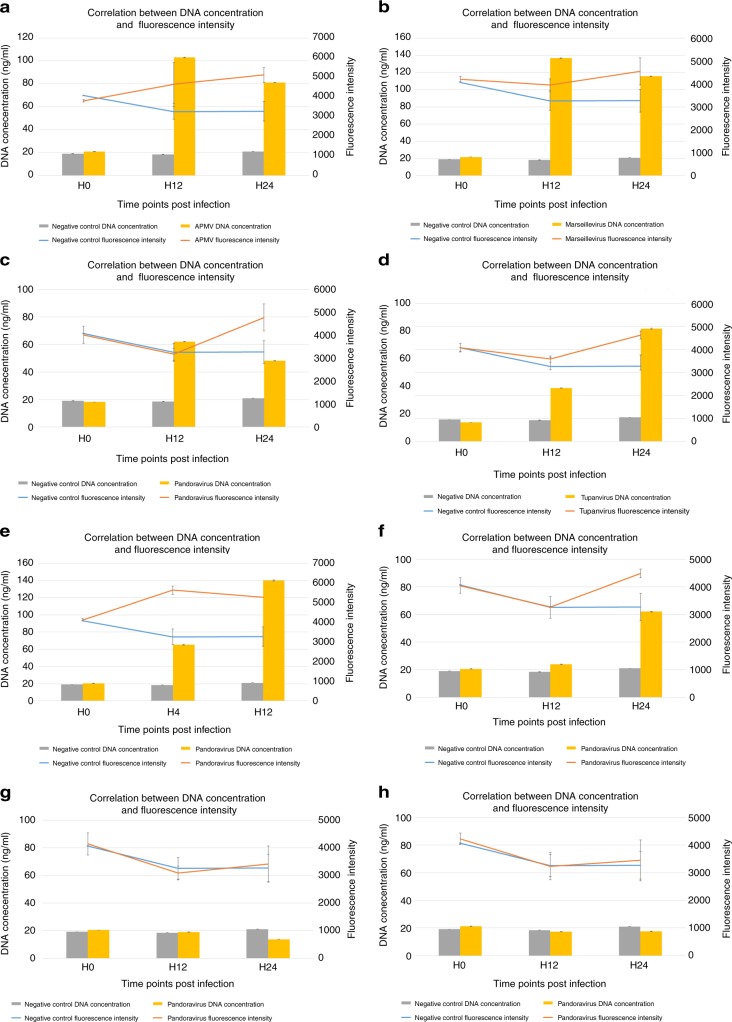
Correlation between fluorescence signal intensity increase and viral DNA replication in *A. castellanii* Neff infected with **a** APMV, **b** Marseillevirus, **c** Pandoravirus, **d** Tupanvirus, **e** Pacmanvirus, **f** Cedratvirus, **g** Faustovirus and **h** Orpheovirus. The mean values and the standard deviations (*n* = 3 independent experiments) are represented (*p*-value of 0.009)

In order to prove that the increased fluorescence intensity is due to viral DNA replication, we performed total quantification of the dsDNA in the cells. The results showed a higher DNA concentration in infected cells than in negative control cells. In the case of *A. castellanii* Neff, this was observed when infected with APMV, Marseillevirus, Pandoravirus, Pacmanvirus, Tupanvirus and Cedratvirus, and no DNA increase was observed with Faustovirus and Orpheovirus (Fig. 3). Experiments were carried out in the starvation medium in which no amebic replication is possible. A positive correlation was found between the fluorescence signal intensity and the total DNA concentration for all tested viruses (Spearman’s *r* = 0.8).

### Automated sample screening and giant virus identification

The automation system coupled with the high-content screening strategy allowed us to monitor the cocultures in real time. Using this system, we were able to consecutively scan multiple plates and monitor the progress of the infection over time. A single plate was screened in less than 30 min. All plates were kept under incubation and protected from any external or cross-contamination during the screen. Samples showing no infection signs were kept under incubation and screened from time to time until complete amoebal encystment.

**Fig. 4:**
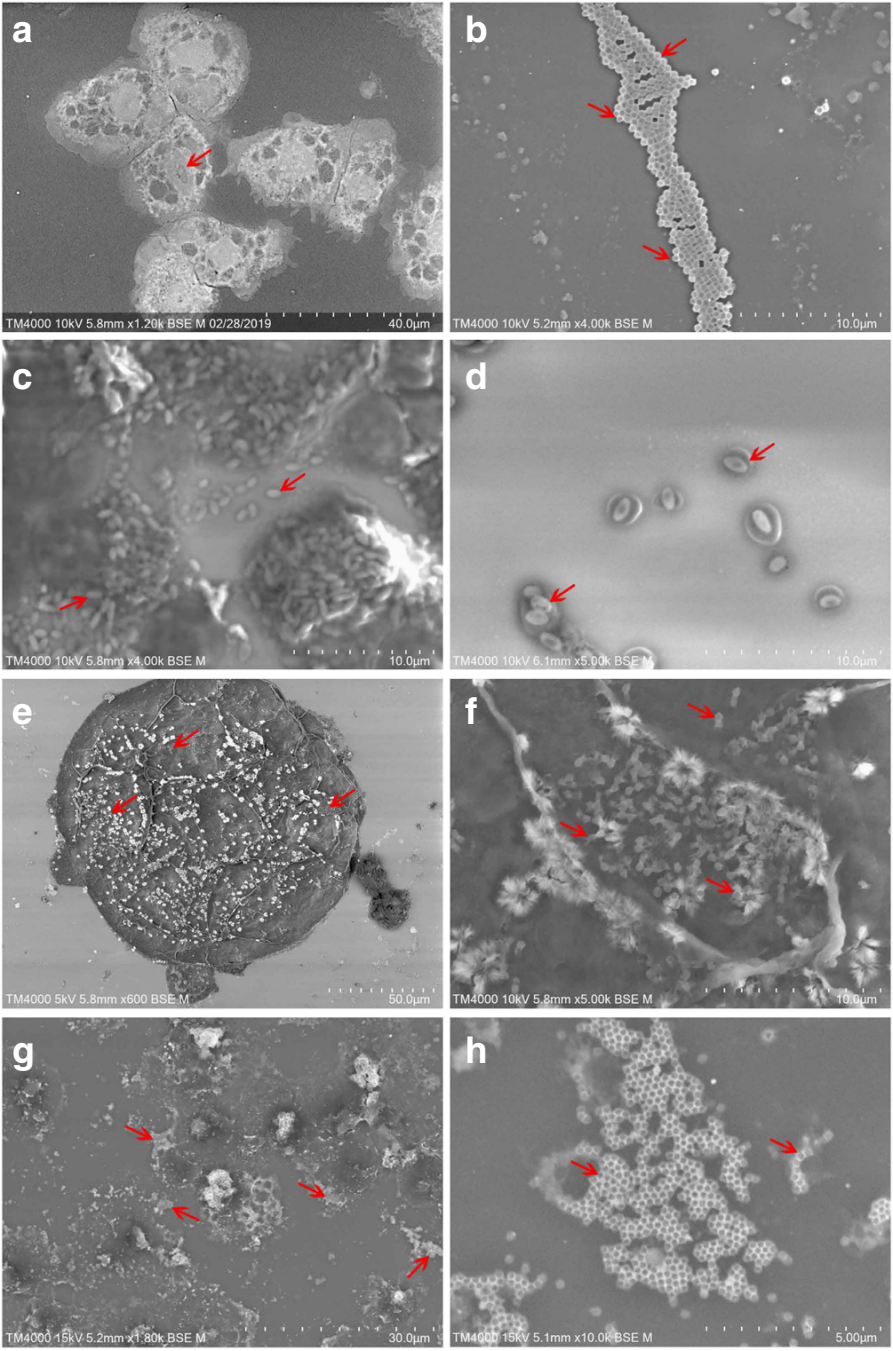
SEM images of culture supernatants showing some of the isolated giant viruses. These photos are generated from our samples using the TM4000 Plus microscope. **a** Uninfected *A. castellanii* Neff (red arrow indicates nucleus). **b** Mimivirus particles showing a typical ~ 650 nm capsid (red arrows). **c**, **d** Pandoravirus particles with their characteristic apical aspect. **e** *A. castellanii* Neff cell with Tupanvirus particles adhered to its surface (red arrows). **f** High-magnification image of **e** showing typical Tupanvirus particles with their characteristic tails (red arrows). **g**, **h** Supernatant of an infected culture showing clusters of Marseillevirus particles with a ~250 nm capsid (red arrows indicate clustered particles). Scale bar and acquisition settings are generated automatically by the SEM on the original micrographs

In cases of complete amoebal lysis or suspected infection, samples were processed for direct identification by SEM using the TM4000 Plus. Some of the positive samples are presented in Fig. 4. A single sample could be scanned within 10min. In parallel, flow cytometry was used for a preliminary identification when complete amoebal lysis was observed. We should mention that the newly synthesized giant viruses were systematically stained with SYBR Green; hence, no additional staining was needed for the gating strategy described in Bou Khalil et al.^32^. As a result, from the samples tested on the 4 amebas, 27 Mimiviruses, 12 Marseilleviruses, 6 Pandoraviruses, 1 Cedratvirus, 1 Pacmanvirus and 4 Faustoviruses were isolated (Supplementary Fig. 3). Among these isolates, only 22 have been previously isolated from these samples using traditional strategies (Supplementary Table 1). All these findings were confirmed by molecular biology using targeted PCR for each specific strain. Three samples showed viral mixtures of Mimivirus and Marseillevirus, Mimivirus and Pandoravirus, as well as Mimivirus and Pacmanvirus. These samples were FACS sorted to separate the viral mixtures^33^.

Then, we compared the isolation rates and time lapse of our new live screening strategy with that of the routinely used isolation technique^32^. All samples were previously tested in our laboratory on the same ameba. The results showed significantly higher isolation rates for our newly implemented isolation strategy on all tested amebas (Table 2).

**Table 2:** Comparison of the isolation rate between the routine isolation strategy and the new live screening strategy

	Isolation rate (%)	
	Flow cytometry strategy	New live screening strategy
*A. castellanii* Neff	20.51	42.31
*V. vermiformis*	2.56	5.12
*A. polyphaga*	3.85	12.82
*A. castellanii* Douglas	1.28	5.13

## Discussion

**Fig. 5:**
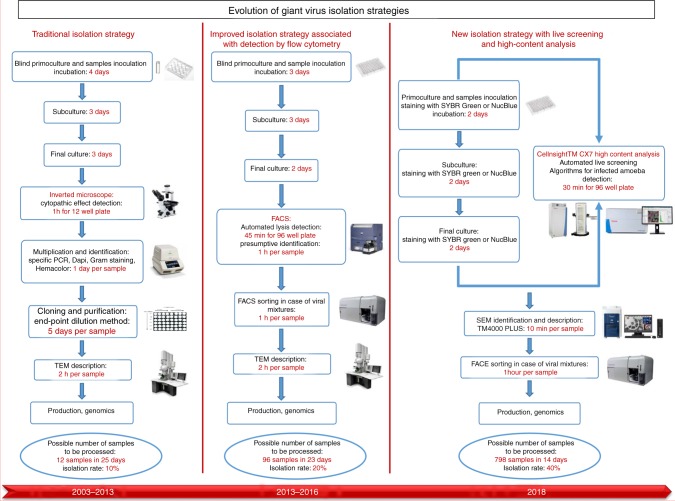
Historic evolution of giant virus isolation strategies since their discovery. Until 2013, giant virus isolation was performed by traditional and operator-dependent techniques using optical microscopy and staining. In 2016, flow cytometry introduced the concept of automated detection and identification. Here, we developed a new isolation strategy introducing new tools allowing the live monitoring of cocultures and high-content analysis for a rapid detection and identification of giant viruses

Many fundamental questions on the ecology, biology, and evolution of giant viruses remain unresolved because of the small number of specimens characterized. Currently, two main tools are used to explore these giant viruses, coculture and metagenomics. Briefly, genomic studies and bioinformatics tools involve tracking, at high speed, potential sequences and genomes of these microbes. These tools have recently made it possible to describe a new family of giant viruses^18^. In parallel, coculture with amoebae is still the key engine for the isolation of giant viruses. This approach has the potential to re-evaluate the incidence and diversity of giant viruses in existing metagenomic data and to introduce novel sequences from poorly sampled viral clades to shed light on the metagenomic dark matter. Furthermore, by isolating the viral particle, we enable full access to the viral components at the genomic, transcriptomic, and proteomic levels and enable the study of virus-host interactions. However, coculture and benchwork techniques are lagging behind the high speed of metagenomic analyses. Therefore, updating the routine isolation strategies is necessary to increase their sensitivity, their isolation speed and rate and to overcome their limitations. For this purpose, we developed a new strategy using high-speed tools allowing live screening and monitoring of the infection during coculture. Infected amebas were detected using specific algorithms targeting the signal and the morphological variations in comparison to the negative controls. A high fluorescence signal was detected at the level of the viral factory. We observed that SYBR Green has some affinity for cytoplasmic and membrane proteins, which resulted in complete diffuse cell staining. Virus factories occupying the cell cytoplasm yielded high-intensity fluorescence signals coming from the viral DNA replication that was confirmed by DNA quantification. In the present work, we adopted the principle of miniaturized coculture optimized by Bou Khalil et al.^32^, but we mainly focused on improving the detection and identification strategies (Fig. 5). We were able to detect the infection at the early stage of primoculture or subculture, which reduced the time required for each enrichment step. This technique allowed the multiparametric detection of infected amebas and the visualization of infected cells. However, few studies have determined the sensitivity and detection limit of systems used for the isolation regarding the viral load in environmental samples, making extended enrichment steps even more critical. This is the case for the prior strategies that relied on blind enrichment steps of three to four days each, followed by a subjective observation under an inverted microscope^26^ or detection by blind flow cytometry where cells are presented as dots and are not actually visualized^32^. This real-time monitoring strategy exhibited higher sensitivity than the old techniques where a very low threshold is needed for detection (3% of infected cells can be detected among all cells and easily differentiated from trophozoites and cysts), which was not the case with the flow cytometry method for which detection is limited to a 50% threshold for lysis^32^.

In addition, the automated system coupled to the high-speed microscope offered many advantages by speeding detection and maintaining cocultures under optimal incubation conditions. Therefore, real-time ameba monitoring allowed us to increase the number and diversity of samples tested (Supplementary Fig. 4), with the Orbiter being able to process up to 40 plates circulating in a closed loop from the Cytomat to the microscope. Moreover, the plates are kept closed during the entire analysis, thus eliminating the risks of any possible contamination or loss of samples. This was a major drawback for the flow cytometry detection method, for which the risk of cross contamination during analysis was very high, and samples were lost afterward^32^. Therefore, duplicate plates were required to continue the analysis. The same was true for agar plate techniques where the risk was higher^31^.

The second major improvement of our method was the use of a tabletop scanning electron microscope for the fast and sensitive characterization of the infectious agents. The TM4000 Plus replaced heavy and time-consuming traditional electron microscopy techniques requiring fastidious sample preparations and hours of imaging (1–2 h for each sample). Here, rapid detection and identification of giant viruses was performed in less than 10 min using the culture supernatants directly for imaging. In addition, we double checked the results by flow cytometry identification and targeted PCR. As a result, with our optimized strategy based on live screening and high-speed detection, we have gained speed, visibility, performance, and sensitivity compared to traditional isolation strategies^26,31,32^ and have successfully tested the same number of samples previously tested over a 6-month period in only two weeks. In addition, we isolated 29 more giant virus strains than those obtained with the traditional isolation technique.

In addition, we are now able to search for nonlytic viruses and detect their presence based on the increase in the fluorescence signal inside the cytoplasm. This was not possible using the flow cytometry method which can only detect lytic agents after cell lysis^32^. In this case, a future approach is to introduce correlative microscopy allowing the detection and characterization of the infectious agent directly by processing infected cell hosts from optical to electron microscopy^34^. Nevertheless, we are aware that our method is currently limited to adherent protists, which makes the flow cytometry an irreplaceable technique because it is applicable for highly motile amebas^22,24^ and able to detect and separate viral mixtures^33^. However, we have increased the number of adherent and axenic amoebae by defining a specific signature or fluorescence profile for each cell type used. Therefore, we can run hundreds of samples on different cell hosts and monitor their profile variations integrated into the software. In addition, this technique showed its efficiency when applied to xenic ameba feeding on bacteria. Although stained bacterial cells were visible outside of the amebas, they were algorithmically excluded from the analysis, and no increase in the associated fluorescence signal was detected in the amebas. A future approach would be the application of this technique to other protists supporting the growth of giant viruses, such as algae^35^ or slow-growing protists, such as *Blastocystis*^36^. To do so, we will monitor cell growth and cell infection at the same time. Finally, this technique is also amenable to many cell lines and could be used in other fields of virology. The ability to simultaneously test a wide variety of hosts and cell samples can help us to further study the infectivity and tropism of giant viruses.

## Methods

### Microorganisms and coculture

For the developmental stage, different amebas were used as cellular supports for the coculture: *Acanthamoeba castellanii* strain Neff 30010 (*A. castellanii* Neff), *Vermamoeba vermiformis* strain CDC19 (*V. vermiformis*), *Acanthamoeba polyphaga* strain Linc AP-1 (*A. polyphaga*), and *Acanthamoeba castellanii* strain Douglas 50370 (*A. castellanii* Douglas). The amebas were cultured in PYG medium (proteose peptone-yeast extract-glucose) at 28 °C for 48 h^26^. They were then harvested, pelleted in PAS (Page’s ameba saline) buffer and resuspended in the starvation medium, as previously described^29,37^. Using kova counting slides (HYCOR Biomedical, Inc., Garden Grove, CA, USA), cell concentrations were adjusted to 4.5 × 10^5^ ameba/ml for *A. castellanii* Neff, *A. polyphaga,* and *A. castellanii* Douglas and to 10^6^ ameba/ml for *V. vermiformis*. Amebas were then transferred to a 48-well plate at a volume of 250 µl per well. Cells were incubated at 32 °C for 1 h prior to infection. Infection was carried out using nine strains of giant viruses: *A. polyphaga* Mimivirus^1^ (APMV), Marseillevirus T19^38^, Faustovirus E12^29^, Pandoravirus massiliensis^39^, Tupanvirus Deep Ocean^7^, Cedratvirus^40^, Orpheovirus IHUMI - LCC2^30^, Pacmanvirus^41^ and a new nonlytic giant virus recently isolated in our lab, Clandestinovirus. The same multiplicity of infection (MOI = 1) was used for each virus. The MOI was determined using the TCID50 method, as previously described^42^. Uninfected cells served as negative controls. DNA staining was carried out using the SYBR™ Green I nucleic acid gel stain or the NucBlue^®^ Live reagent (Hoechst 33342) (Molecular Probes, Life Technologies, USA). The viability with each DNA stain was assessed for all four amebas, where we performed daily screenings on ameba to search for a toxic effect when cells were exposed to these dyes. Minimal concentrations granting optimal ameba viability were chosen for all experiments. The concentration for SYBR Green was set to 2 × 10^−4^ dilution of the commercial stock solution, and the concentration for NucBlue was 4 ng/ml. We then studied the infectivity and fitness of certain giant viruses on the chosen panel of amebas over a period of 48 h.

### Configuration of the microscope to detect infected ameba

Cell imaging and analysis was performed on an integrated imaging platform consisting of an automated incubator Cytomat TM 2C-LIN (Thermo Scientific), linked to a robotic arm Orbitor™ RS Microplate mover (Thermo Scientific), together targeting the automated CellInsight CX7 High-Content Screening Platform microscope (Thermo Scientific).

Thermo Scientific™ Momentum 5.0.5 software supervised the time of incubation, plate handling and screens at fixed time points. The imaging parameters, such as autofocus settings, exposure time and cell analysis, were predefined in HCS Studio 3.0 software (Thermo Scientific). HCS Studio 3.0 delivers an embedded image algorithm for cell identification and on-the-fly multiparametric analysis. We scanned 1000–2000 cells per well using the SpotDetector BioApplication. The contrast was sufficient on both fluorescence and brightfield images to allow intensity-based detection of amebas. Outliers (ameba aggregates, noncellular objects and extreme intensity values as a result of nonspecific staining) were removed algorithmically.

We investigated cellular changes or signal changes between negative controls and infected cells. To this end, we measured the shape index (expressed as 4*perimeter^2^/π*area) and the DNA content as the average and/or the total intensity of the SYBR Green or NucBlue fluorescent probes. A cytopathic effect, as well as a variation in the intensity of the fluorescence signal (decrease or increase), was followed throughout the infection process. We reported the mean values per well. Exposure times for the 386 nm, 485 nm and brightfield channels were adjusted so that the fluorescent or optical density signal reached 50% of the dynamic range of the 14-bit camera in the negative control. Image acquisition was performed at ×20 (NA .70) and ×40 magnifications. Regarding the screening strategy, two simultaneous upper limit settings were fixed: scanning 1000 cells per well and/or a number of images not exceeding 20 images per well. The plates were scanned every 4 h over a period of 48 h pi. Different areas were programmed to be swept at each moment in order to screen the entire well. All experiments were performed in triplicate.

### System sensitivity and detection limit

After configuring the system, we tested its sensitivity for low viral MOIs mimicking the viral load in environmental samples. For this, *A. castellanii* Neff, at a concentration of 4.5 × 10^5^ ameba/ml, was infected with APMV at different MOIs (up to an MOI of 10^−10^). The infection was monitored over a period of 5 days to determine the detection threshold (number of infected cells detected in a well).

### Artificial sample detection

For the validation stage, a blind test was performed using several samples of water and sewage. Some of the samples were artificially contaminated separately with the following giant viruses: Mimivirus, Marseillevirus, Pandoravirus, Faustovirus, and Tupanvirus. We contaminated 5 out of 12 samples previously tested as negative using our standard isolation procedure^32^. Note that the CX7 microscope operator for the detection step was ignorant of the number and identity of the contaminated samples.

The coculture consisted of three steps of enrichment^32^: the primoculture, the subculture and the final culture. Each step was assessed with a combined real-time acquisition and analysis for 48 h. Uninfected amebas were used as negative controls. For bacterial and fungal growth inhibition, antibiotic and antifungal mixtures containing 10 µg/ml vancomycin, 10 µg/ml imipenem, 20 µg/ml ciprofloxacin, 20 µg/ml doxycycline and 20 µg/ml voriconazole were added to the cocultures as previously described^26,37^. We applied the same screening strategy detailed above for screening, imaging and cell analysis.

### Xenic culture model

In a second step, we tested the possibility of using xenic ameba feeding on bacteria. The objective was to identify possible interference between the fluorescence signal coming from the bacteria and that coming from the viral replication. To do so, we used a model of xenic ameba coculturing *A. castellanii* Neff with live *E. aerogenes* at a concentration of 10^7^ bacteria/ml in PAS buffer. Amebas were then infected with APMV at an MOI of 1. SYBR Green was used for DNA staining, and the culture was monitored for 48 h. Uninfected ameba feeding on *E. aerogenes* served as a negative control.

### Validating the correlation between fluorescence intensity signal and viral replication

This step was processed to confirm that the variation in signal intensity was actually due to viral replication. We performed a quantification of the total dsDNA of *A. castellanii* Neff infected with APMV, Marseillevirus, Pandoravirus, Tupanvirus, Pacmanvirus, Cedratvirus, Faustovirus and Orpheovirus. Uninfected amebas were used as negative controls. Both infected and uninfected wells were used, and the cells were pelleted by centrifugation at 2000 rpm for 10min and then resuspended in an equal volume of starvation medium. DNA was extracted using an EZ1^®^ DNA Tissue Kit (Qiagen GmbH, Hilden, Germany). Following the extraction, DNA was quantified using the Qubit™ dsDNA HS Assay Kit (catalog number Q33231, Invitrogen) and the Qubit 4 Fluorometer (Invitrogen) per the manufacturer’s instructions. The experiment was performed in triplicate at 0 h, 12 h, and 24 h pi for all giant viruses except Pacmanvirus, which was quantified at 0 h, 4 h, and 12 h pi.

### High-throughput automated sample screening

For high-throughput detection, all conditions of the coculture steps were optimized and adapted for the use of 96-well microplates. The ameba cell volume was optimized to 200 µl per well at the same concentrations described above for each ameba. Fifty microliters volume of each sample was used for the inoculation. We integrated the automation system described above to perform high-throughput automated screening for giant virus isolation in environmental samples. We used a set of frozen environmental samples from our collection: 38 sewage and stool samples (from Oran-Algeria, Algiers-Algeria, Oued-Algeria, Var-France and Marseille-France), 28 swimming pool water, seawater and lake water samples (from Oran-Algeria, La Ciotat-France, Var-France, Riboux-France, Saint Tropez-France and Port Saint Louis-France) and 12 samples of soil, fungi and algae (from Var-France and La Ciotat-France). These samples had previously been tested in our laboratory with the traditional isolation strategy using flow cytometry^32^. All samples were cultured on *A. castellanii* Neff, *V. vermiformis*, *A. polyphaga* and *A. castellanii* Douglas. Each plate was associated with an acquisition protocol for high-content analysis. A specific ID and incubation pattern were attributed to each plate. The same screening strategy detailed above was used for every enrichment step. The presence of giant viruses was detected prior to host cell lysis or after cell lysis, where it is important to note that the cytopathic effect is sample- or virus-dependent.

### Identification and characterization of giant virus isolates

Following complete amoebal lysis, we developed a new strategy for the identification process. After infection monitoring, we first used a new tabletop scanning electron microscope (SEM) TM4000 Plus from Hitachi for presumptive identification of giant viruses. This microscope has the ability to observe the sample at low pressure under a vacuum (10^0^ Pa to 10^1^ Pa) to reduce the charge increase on the specimen surface by the irradiated electrons. The evacuation time after loading the sample into the SEM chamber is less than 2min, which is much faster than conventional SEMs. Samples showing potential infection were directly processed on the TM4000. The culture supernatant was cytocentrifuged directly on slides at 800 rpm for 10 min^26^, stained with PTA (phosphotungstic acid, Sigma-Aldrich, Germany) and then directly observed under the SEM microscope. In parallel, the process of identification using flow cytometry described by Bou Khalil *et al*. was followed as a control^32^. Briefly, wells exhibiting cell lysis were pipetted and centrifuged to eliminate cell debris (2000 rpm for 10 min). The supernatant was diluted to 1/10^4^ in PAS buffer. Samples were then directly processed by flow cytometry without additional staining. In the case of viral mixtures, FACS sorting was performed to separate giant viruses^33^. After detection and identification, we verified the isolates by targeted PCR^26,33^.

### Statistics and reproducibility

We used R software, a language and environment for statistical computing and analysis version 3.5.1. Spearman correlations were used to investigate possible links between fluorescence signal intensity and total DNA quantification. A *p*-value was also generated to identify significant differences between control and infected cell fluorescence intensities, shape index and total count. A *p*-value of <0.05 was considered statistically significant. Experiments were performed in triplicate.

### Reporting summary

Further information on research design is available in the Nature Research Reporting Summary linked to this article.

## Supplementary information

Supplementary Information

Reporting Summary

## Data Availability

The authors declare that all data supporting the findings of this study are available within the paper and its supplementary data.
