# Supplementary material for: High-throughput isolation of giant viruses using high-content screening
Source: Commun Biol. 2019 Jun 19;2:216. doi: 10.1038/s42003-019-0475-6 (PMC6584669; doi:10.1038/s42003-019-0475-6)
Supplement: Supplementary file 1 — Supplementary Information [file 42003_2019_475_MOESM1_ESM.pdf]

Supplementary Figures

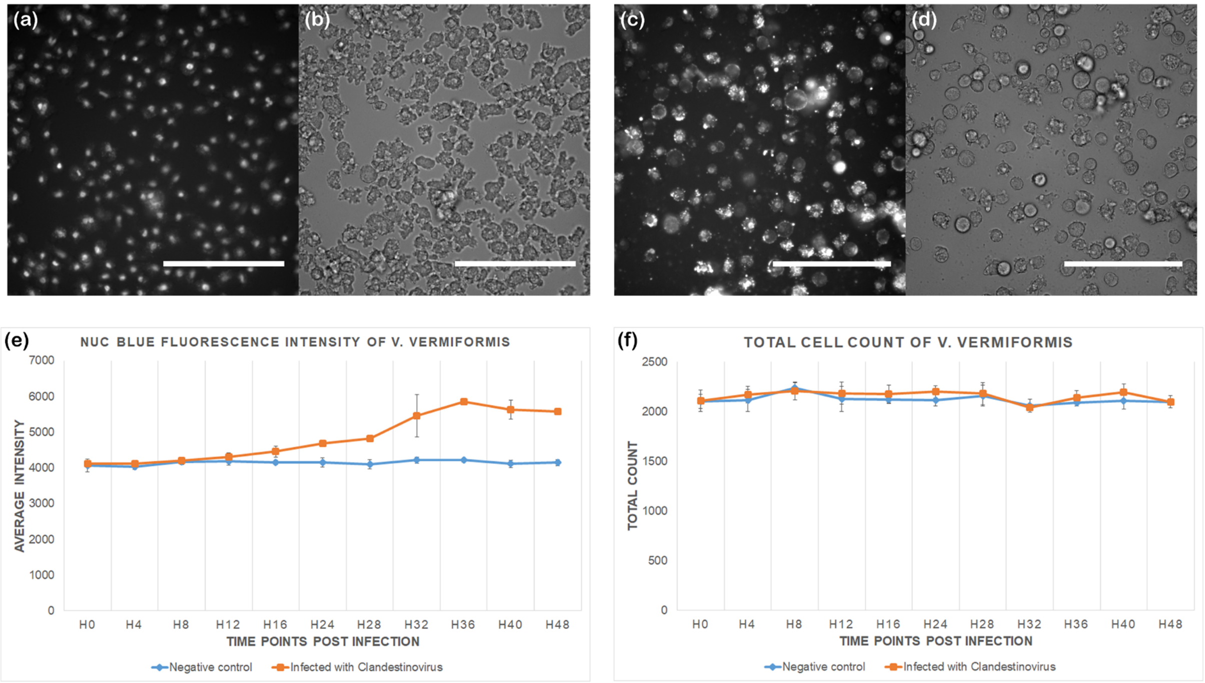

Supplementary figure 1: ***V. vermiformis*** infected with a non-lytic Clandestinovirus analyzed by High Content Screening. Cells are stained with Nuc Blue. (a) and (b) Nuc Blue and brightfield channels respectively for the negative control at 48 h pi. (c) and (d) Nuc Blue and brightfield channels respectively for Clandestinovirus at 48 h pi (40x magnification). The scale bars indicate 50 µm. (e) Nuc Blue average fluorescence intensity, (f) Total cell count. The mean values are represented for each parameter. Error bars represent standard deviations. Experiments were performed in triplicate (n =3).

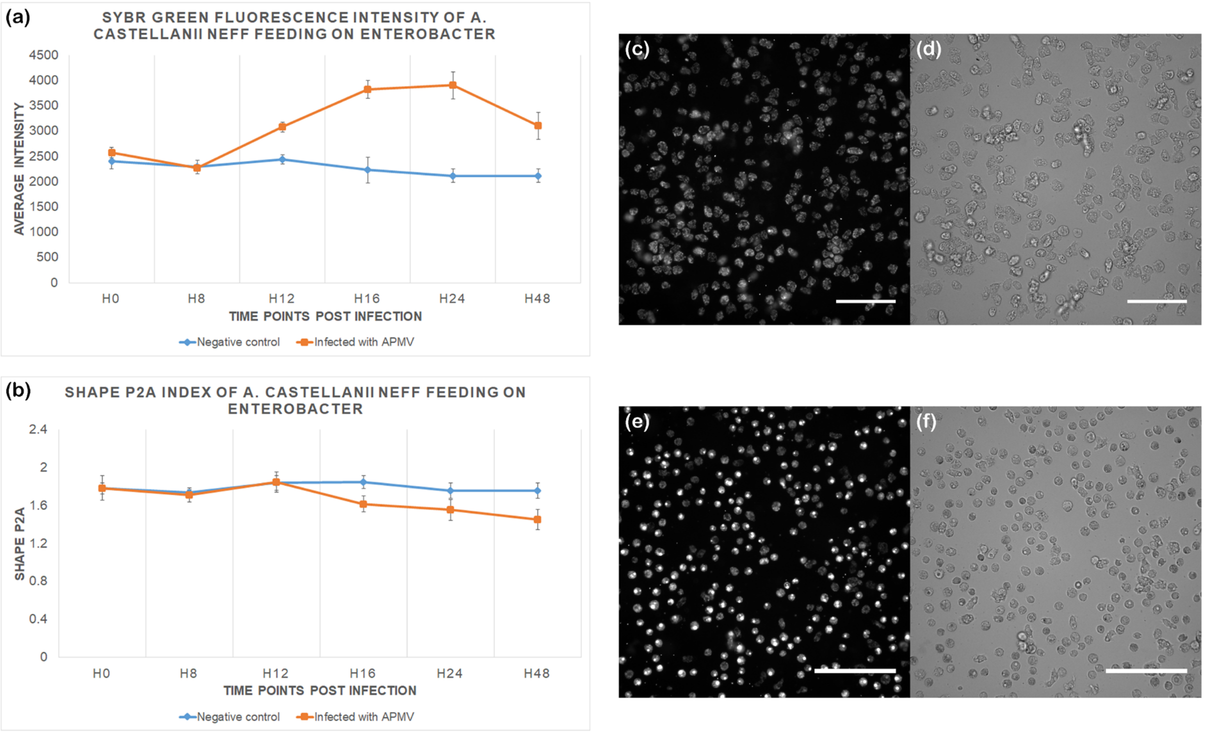

Supplementary figure 2: Xenic culture of *A. castellanii* Neff feeding on *Enterobacter aerogenes* analysed by High Content Screening. Cells are stained with SYBR Green. Uninfected *A. castellanii* Neff feeding on bacteria served as a negative control. (a) SYBR Green Average Intensity, (b) Cell Shape P2A Index. The mean values are represented for each parameter. Error bars represent standard deviations. (c) and (d) SYBR Green and brightfield channels respectively for the negative control at 16 h pi. (e) and (f) SYBR Green and brightfield channels respectively for APMV at 16 h pi (40x magnification). The scale bars indicate 100 µm. Experiments were performed in triplicate (n = 3)

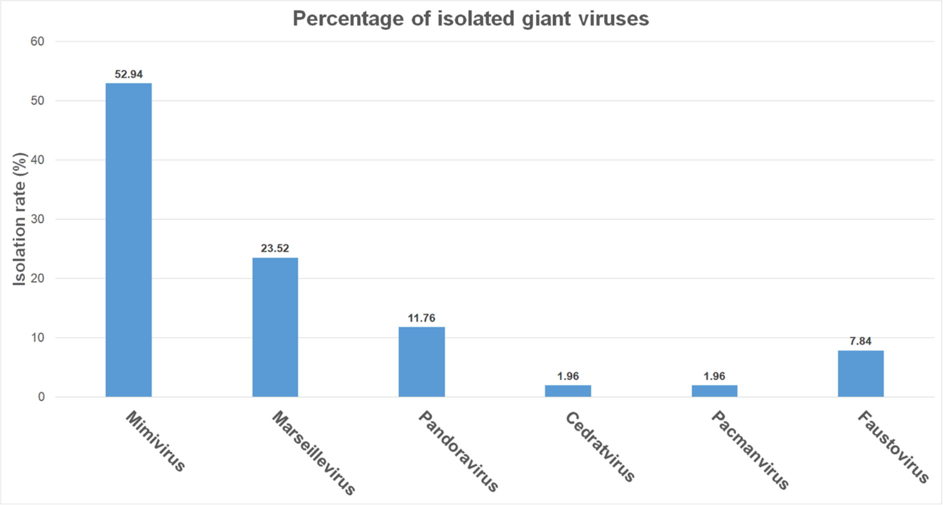

Supplementary figure 3: Percentage of giant viruses isolated by the new High Content Screening strategy. In this figure, we present the giant viruses isolated from the environmental samples tested by our new isolation strategy (n = 80).

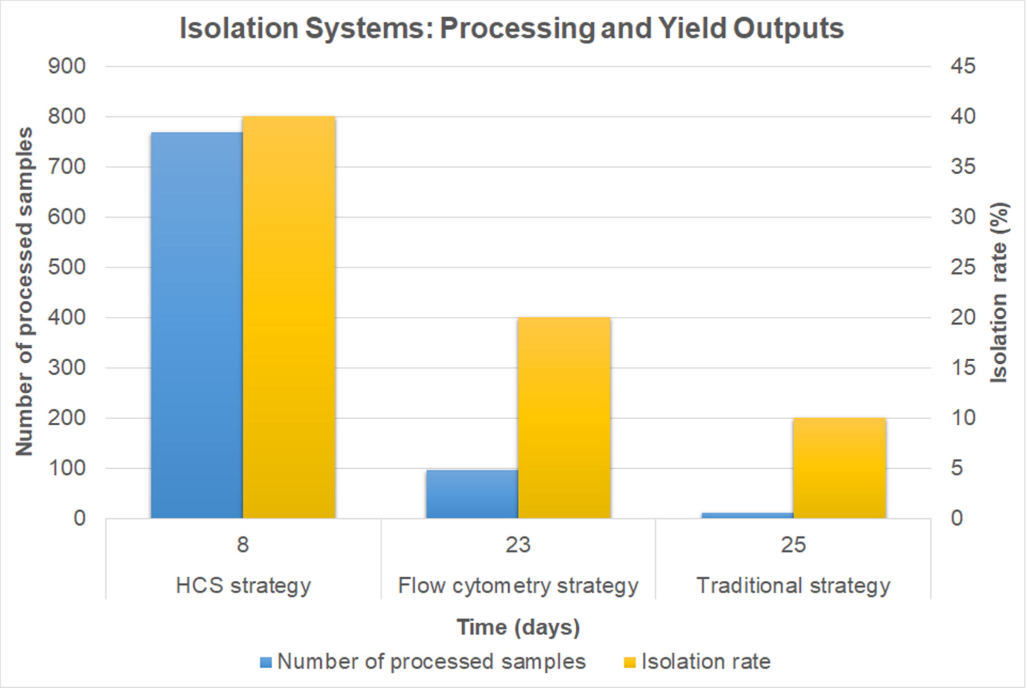

Supplementary figure 4: Processing and yield outputs of the different isolation systems (n = 80).

Supplementary Tables

Sample type	Country	Code	Traditional isolation strategy				New live screening strategy			
			***A. castellanii* Neff**	***V. vermiformis***	***A. polyphaga***	***A. castellanii* Douglas**	***A. castellanii* Neff**	***V. vermiformis***	***A. polyphaga***	***A. castellanii* Douglas**
Sewage	Algeria	S3					Mimivirus			
	Algeria	S4	Marseillevirus		Mimivirus		Marseillevirus		Mimivirus	
	Algers-Algeria	S7	Marseillevirus		Mimivirus		Marseillevirus		Mimivirus	
	Algers-Algeria	S8					Mimivirus			
	Algers-Algeria	S10					Marseillevirus			
	Oran-Algeria	S11	Marseillevirus + Mimivirus				Marseillevirus + Mimivirus			
	Oran-Algeria	S12					Mimivirus			
	Oran-Algeria	S13					Mimivirus		Mimivirus	Mimivirus
	Oran-Algeria	S15	Mimivirus				Mimivirus			
	Oran-Algeria	S17	Marseillevirus	Faustovirus			Marseillevirus	Faustovirus		
	Oran-Algeria	S18	Marseillevirus				Marseillevirus			
	Oran-Algeria	S19	Pacmanvirus				Marseillevirus+ Pacmanvirus		Mimivirus	
	Oran-Algeria	S20	Marseillevirus				Marseillevirus		Mimivirus	
	Marseille-France	S41	Pandoravirus				Pandoravirus			
	Algeria	A2	Mimivirus		Mimivirus	Mimivirus	Mimivirus	Faustovirus	Mimivirus	Mimivirus
	Algeria	A3	Pandoravirus				Pandoravirus			
	Algeria	A6					Mimivirus		Mimivirus	
Wild boar stool	Var-France	S24							Mimivirus	
	Var-France	S25							Mimivirus	
	Var-France	S27					Pandoravirus			
Pigeon saddles	France	S45					Mimivirus			
	Algeria	A5						Faustovirus		
Stool of house sparrows	Marseille-France	S50					Pandoravirus			
	Marseille-France	S53	Pandoravirus							
Lake water	Algeria	S5					Marseillevirus			
	Oran-Algeria	S58					Marseillevirus			
Rain water	La Ciotat-France	S21	Pandoravirus				Pandoravirus			
Pool water	Algeria	A13					Mimivirus			
Tank water	Riboux-France	S37					Marseillevirus			
Drainage water	Algeria	S55					Mimivirus		Mimivirus	Mimivirus
Soil	Oued-Algeria	A1				Mimivirus				Mimivirus
Gutter	Algeria	S57		Faustovirus			Marseillevirus	Faustovirus		
Compost	La Ciotat-France	S35	Pandoravirus				Pandoravirus + Mimivirus			
	France	S38					Marseillevirus			
Alpova sp.	Var-France	S29	Cedratvirus				Cedratvirus			

Supplementary Table 1: Giant virus isolates from environmental samples using the traditional flow cytometry isolation strategy and the new HCS isolation strategy.
